# Stability bearing capacity of concrete filled steel tubular columns subjected to long-term load

**DOI:** 10.1038/s41598-023-37596-x

**Published:** 2023-06-27

**Authors:** Xiuying Lai, Huabin Gao, Zhen Yang, Juan Zheng, Zhaoyu Chen, Fuming Lin

**Affiliations:** 1 School of Civil Engineering, Putian University, Putian, 351100 China; 2 Xiamen Iport Group Co. Ltd, Xiamen, 361000 China; 3 Network and Information Center, East China Jiaotong University, Nanchang, 330013 China; 4 Fujian Architectural Design and Research Institute Co., LTD, Fuzhou, 350000 China; 5 Xiamen Baicheng Building Materials Co., Ltd. Xiamen, Fujian, 361021 China

**Keywords:** Civil engineering, Composites

## Abstract

Concrete-filled steel tube (CFST) are commonly used in modern building and bridge applications. Despite their popularity, studies on the investigation of the influence of long-term load on the stability bearing capacity of such elements are scarce. This study investigates how the key parameters including slenderness ratio (*λ*), axial load ratio (*m*), and eccentricity ratio (*e*/*r*) affect the stability bearing capacity of a CFST column under sustained load. Twenty three CFST columns were fabricated to investigate the effect of long-term load on the stability bearing capacity. Fourteen specimens were subjected to constant compressive loading for 462 days and then tested for failure. The remaining 9 were companion load-free specimens. A three-stage finite element method was used to predict the stability bearing capacity after creep. The results indicate that the stability bearing capacity of CFST columns decrease after being subjected to long-term load. Both the experimental and numerical results indicated that the load of steel tube for long-term load specimens reaching up to the elastic–plastic and plastic process was lower than that of the load-free specimens. Moreover, the corresponding strain of the creep specimens was greater than that of the load-free specimens when the member reached the maximum load. Benchmarking analyses have shown that the creep reduction coefficient (*k*_cr_) proposed for CFST columns can be used to predict the reduction of stability bearing capacity after creep. Furthermore, a collected database comprising 49 CFST specimens subjected to long-term load was used to investigate the proposed formulae for *k*_cr_. The results show that the formulae were consistent with the experiment results.

## Introduction

Concrete filled steel tube (CFST) members are efficient compression structural components used in civil engineering structures, such as arch bridges and columns used in buildings^1–6^. In contrast to steel or reinforced members, the behavior and strength of CFST members are significantly improved owing to interactions between the steel tube and the concrete infill^6^. Moreover, the steel tube can serve as a permanent formwork placing concrete, which facilitates the construction process. With the continuous improvements in material and construction techniques, the span of newly built CFST arch bridges has been increasing, with the longest span reaching 575 m^5^.

With the longest arch span length record being constantly broken, the buckling of the arch rib has gradually become a prominent problem owing to the increasing slenderness ratio. Moreover, the creep effects on the CFST arch rib are other prominent issues encountered with the increasing self-weight of CFST arch bridges.. When checking the stability bearing capacity of a slender CFST arch rib, it can be considered as equivalent to a slender column^6^. Therefore, most studies were conducted on the creep behaviors for CFST columns^7–14^. These studies focused on the creep development and predicted models of CFST columns with normal concrete, recycled aggregate concrete, expansive concrete, and reinforced concrete. In contrast, experimental studies have investigated the stability ultimate bearing capacity of CFST columns subjected to long-term load. Only several theoretical discussions and deduced formulations have been conducted to investigate the ultimate bearing capacity of CFST columns subjected to long-term load. All these studies have demonstrated that the stability bearing capacity of CFST columns decreases because of the creep behavior^15–20^. Further, numerical calculation methods have been used to analyze the stability bearing capacity of CFST columns affected by long-term load, and consequently, the calculation formulae of creep reduction coefficient of CFST column has been derived^21–23^. Tan et al.^24^ stated that the stability bearing capacity of CFST columns is not reduced after being subjected to long-term load. The results of refs. 25 and 26 have indicated a decrease in the stability bearing capacity of CFST columns because of the long-term load. In contrast, certain scholars^21,27–29^ have concluded that the compressive strength of CFST is improved when subjected to long-term load.

Despite several useful studies on the stability bearing capacity of CFST columns affected by long-term load, most of the research results have been obtained based on numerical analysis. Only one study 24 experimentally examined the bearing capacity of 22 CFST creep members. However, the maximum slenderness ratio was only 16, which cannot reflect the creep effect of CFST columns with large slenderness ratio. Thus, there is a lack of in-depth research and reasonable explanation on the mechanism of the effect of long-term load on the stability bearing capacity of CFST columns. Thus, this study conducted a creep experiment to investigate the influence of the stability bearing capacity of CFST columns after being subjected to long-term load. Consequently, a three-stage finite element method was proposed to predict the stability bearing capacity after creep. Thereafter, a parametric analysis considering the key factors was conducted, and a calculation method or the creep influence coefficient was developed to estimate the long-term responses of the stability bearing capacity of CFST columns.

The remainder of this paper is organized as follows. Section "Experimental Program" presents the design scheme of this test and the fabrication of all experimental specimens. Further, the test setup and instrumentation are also introduced in this section. Section "Test Results and Discussion" presents the failure mode and the effects of slenderness, axial load, and eccentricity ratios on the stability bearing capacity of CFST columns. Thereafter, Section "Finite-Element analysis" presents the calculation method of finite-element analysis for the creep specimens and the parameter analysis for further creep members. In addition, the practical algorithms and suggestions for calculating the influence of the long-term load subjected to CFST columns are proposed in this section. Finally, Section "Summary and Conclusions" presents the study results obtained from the test performed along with other test results. The limitations of this study and future research prospects are also presented in this Section.

## Experimental program

Creep experiments were first performed with 462 days of being subjected to long-term load. Thereafter, compression tests were conducted on the creep specimens still subjected to long-term load and companion load-free specimens. The literature 15–16, 30 shows that the slenderness ratio (*λ*), axial load ratio [*m*, the ratio of the long-term axial load (*N*_L_) to the design axial compressive strength (*N*_0_)], and eccentricity ratio (*e*/*r*) of CFST specimens are the most important parameters affecting the stability bearing capacity of CFST members under long-term load. Therefore, these were selected as the main design parameters in the test.

### Test specimen

A total of 23 specimens of CFST columns were fabricated to investigate the ultimate bearing capacity subjected to long-term load. The specimens were divided into four groups. Specimens of Groups I, II, and III were subjected to long-term load, whereas the load-free specimens in Group IV were considered as the comparison specimens. Further, the shrinkage strain was measured simultaneously. The outer diameter of all the specimens (*D*) was 140 mm with the height in range of 350–2100 mm (i.e., *L* = 350, 700, 1050, 1400, 1750, and 2100 mm). The wall thickness of the steel tube was 2 mm, with a steel content of 0.057. The slenderness ratio (*λ* = 4*L*/*D*) of the specimens varied in the range of 10–60, as presented in Table 1. For specimens in Group I, the slenderness ratio (*λ*) was considered as the specimen parameter, where the λ was set to 10, 20, 30, 40, 50, and 60. The axial load ratio (*m*) was considered as the specimen parameter in Group II, with *m* set as 0.1, 0.2, 0.3, 0.4, 0.5, and 0.6. Further, the eccentricity ratio (*e*/*r*) of long-term load was considered as the specimen parameter in Group III. Finally, Group IV comprised the comparison specimens that were not subjected to the long-term load. The specific parameters are listed in Table 1. The number of specimens in Groups I, II, and III in the table are described as follows: CFT-30–0.3–0.1-CR represents CFST creep specimens with a slenderness ratio (*λ*) of 30, an axial load ratio (*m*) of 0.3, and an eccentricity ratio (*e*/*r*) of 0.1. The number of the specimens in Group IV was described as follows: CFT-10–0-0-SH, which indicates the CFST shrinkage specimen with a slenderness ratio (*λ*) of 10 and an axial load ratio (*m*) of 0 without being subjected to long-term load. In this study, a concrete grade of C50 was used in the test, and the age of concrete when applying the long-term load was 7 days. In the table, the axial load ratio is defined as *m* = *N*_L_/*N*_0_, where *N*_L_ is the value of the long-term load applied to the specimen, and *N*_0_ is the design ultimate bearing capacity of the specimen. Here, *N*_0_ is predicted using a formula as per the national standard GB 50,923–2013^31^ for concrete-filled steel tube arch bridges. It is expressed as *N*_0_ = *k*_3_(1.14 + 1.02*ξ*_0_)(1 + *α*)*f*_cd_*A*_c_; where *f*_cd_ is the compressive strength of concrete, *A*_c_ is the cross-section area of core concrete, *ξ*_0_ = *αf*_y_/*f*_cd_ is the confining coefficient, *α* is the steel ratio, *f*_y_ is the yield strength of steel tube, *k*_3_ is the conversion coefficient of axial compressive strength. Further, *N*_ue_ is the tested ultimate bearing capacity of the specimen, and *N*_uc_ is the finite element calculated ultimate bearing capacity of the specimen.

**Table 1:** list of test specimens.

NO	Specimens	*D* × *t*_*s*_ × *L*(mm)	*λ*	*m*	*e*/*r*	*N*_L_ /kN	*N*_ue_ /kN	*N*_uc_ /kN	*N*_ue_/ *N*_uc_
I	CFT-10–0.5–0-CR	140 × 2 × 350	10	0.5	0	500	1165	1179	0.988
	CFT-20–0.5–0-CR	140 × 2 × 700	20	0.5	0	500	1129	1145	0.986
	CFT-30–0.5–0-CR	140 × 2 × 1050	30	0.5	0	500	1045	1065	0.981
	CFT-40–0.5–0-CR	140 × 2 × 1400	40	0.5	0	500	914	927	0.986
	CFT-50–0.5–0-CR	140 × 2 × 1750	50	0.5	0	500	798	790	1.010
	CFT-60–0.5–0-CR	140 × 2 × 2100	60	0.5	0	500	694	679	1.022
II	CFT-30–0.1–0-CR	140 × 2 × 1050	30	0.1	0	100	1074	1096	0.980
	CFT-30–0.2–0-CR	140 × 2 × 1050	30	0.2	0	200	1066	1088	0.980
	CFT-30–0.3–0-CR	140 × 2 × 1050	30	0.3	0	300	1067	1075	0.993
	CFT-30–0.4–0-CR	140 × 2 × 1050	30	0.4	0	400	1059	1069	0.991
	CFT-30–0.6–0-CR	140 × 2 × 1050	30	0.6	0	500	1044	1057	0.988
III	CFT-30–0.3–0.1-CR	140 × 2 × 1050	30	0.3	0.1	300	946	912	1.037
	CFT-30–0.3–0.2-CR	140 × 2 × 1050	30	0.3	0.2	300	695	745	0.933
	CFT-30–0.3–0.3-CR	140 × 2 × 1050	30	0.3	0.3	300	648	717	0.904
IV	CFT-10–0-0-SH	140 × 2 × 350	10	0	/	/	1162	1176	0.988
	CFT-20–0-0-SH	140 × 2 × 700	20	0	/	/	1139	1152	0.989
	CFT-30–0-0-SH	140 × 2 × 1050	30	0	/	/	1089	1101	0.989
	CFT-30–0-0.1-SH	140 × 2 × 1050	30	0	0.1	/	979	911	1.075
	CFT-30–0-0.2-SH	140 × 2 × 1050	30	0	0.2	/	846	892	0.892
	CFT-30–0-0.3-SH	140 × 2 × 1050	30	0	0.3	/	778	717	1.085
	CFT-40–0-0-SH	140 × 2 × 1400	40	0	/	/	1014	1025	0.989
	CFT-50–0-0-SH	140 × 2 × 1750	50	0	/	/	940	948	0.992
	CFT-60–0-0-SH	140 × 2 × 2100	60	0	/	/	859	864	0.994

**Figure 1:**
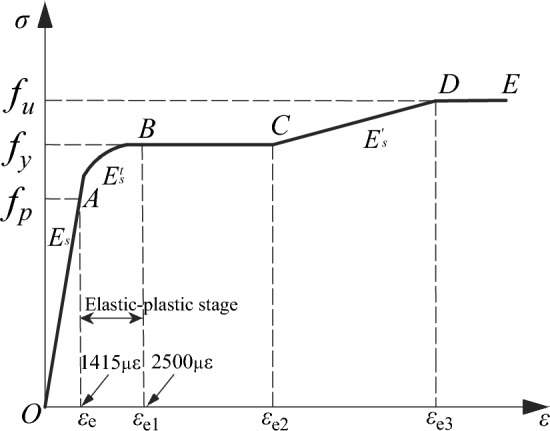
Material property curve of steel tube.

A seamless steel tube with a strength grade of Q235 was used in this test, with its measured yield strength (*f*_*y*_), tensile strength (*f*_*u*_), and elastic modulus (*E*_*s*_) being 350 MPa, 423 MPa, and 198 GPa, respectively. As shown in Fig. 1, the stress (*σ*)—strain (*ε*) curve of the steel tube can be divided into 5 stages:. Elastic (OA), elastic–plastic (AB), plastic (BC), hardening (CD), and secondary plastic flow (DE) stages. The measured proportional limit strain (*ε*_*e*_) and yield limit strain (*ε*_e1_) of the steel tube were 1415 µɛ and 2500 µɛ, respectively.

**Figure 2:**
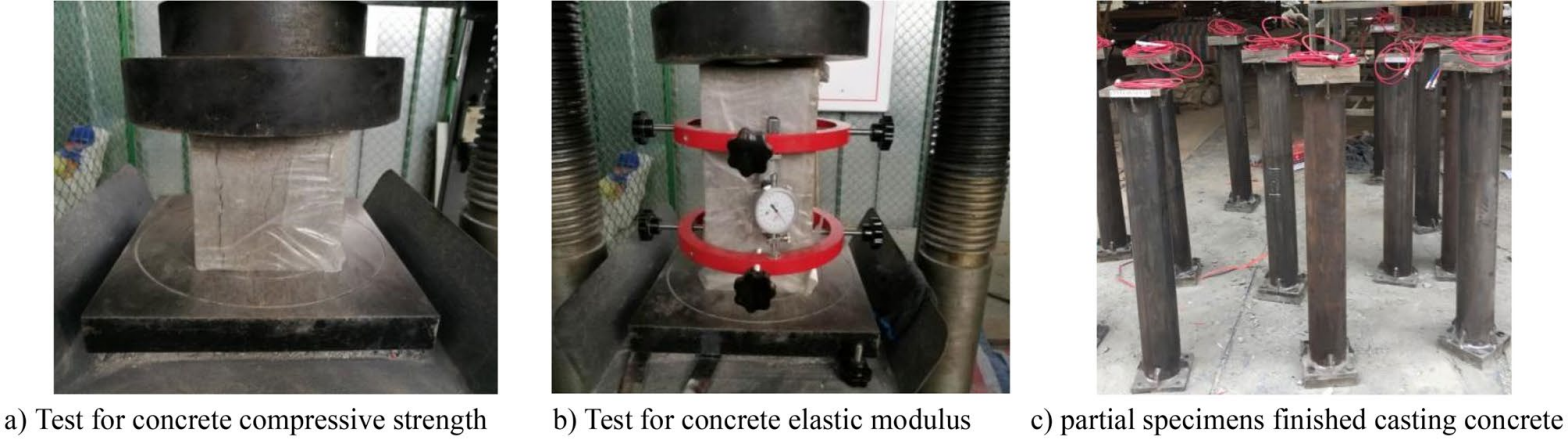
Partial photos for test.

Table 2 summarizes the mixture proportions of the concrete infill. These include the proportions of the cement (P.O.42.5 ordinary Portland cement), coarse aggregate (granite stone, 5–10 mm in diameter), sand, water, superplasticizer, and fly ash. Table 3 summarizes the concrete grade, concrete compressive strength (*f*_*cm*_), and elastic modulus (*E*_*c*_) measured at 7 and 462 days. The concrete grade represents the nominal 28-day concrete compressive strength (*f*_*cu,k*_) in MPa, whereas the *f*_*cm*_ represents the measured value. Here, *f*_*cm*_ was determined as the average value measured from three 150-mm concrete cubes as per the “China Standard for Test Methods of Concrete Physical and Mechanical Properties”^32^. To ensure that the concrete infill is sealed, a 190 × 200 mm steel plate of 20 mm thickness was welded to the bottom end of each hollow steel tube prior to the casting of concrete, as shown in Fig. 2. During casting, the specimens were maintained in a vertical position and the steel tubes were maintained in an ungreased state to reflect the common site practice. Further, all the specimens were cast on the same day in one batch. Moreover, the concrete core was cast slightly higher than the steel tube to avoid the presence of gaps between the concrete core and the top steel plate before welding the top steel plates. Immediately after the concrete pouring, the top surfaces of the specimens were tightly wrapped with plastic films to reflect the real situation wherein the concrete core remained sealed during construction and while in service. The plastic films were removed after 1 day and the top surfaces of the CFST specimens were grounded plain and smoothed. Thereafter, a 190 × 200 mm steel plate of 20 mm thickness was welded to the top of the steel tube to seal the concrete immediately after completing the smoothing operation.

**Table 2:** Concrete mix proportion for CFST specimens.

Concrete grade	Cement (kg/m^3^)	Coarse aggregate (kg/m^3^)	Sand (kg/m^3^)	Water (kg/m^3^)	Fly ash (kg/m^3^)	Superplasticizer (kg/m^3^)
C50	443	1087	638	177	74	5.9

**Table 3:** Measured values of concrete properties at different ages.

Concrete grade	Compressive strength [*f*_*cm*_(MPa)]		Elastic modulus [*E*_*c*_(GPa)]	
	7d	462d	7d	462d
C50	49.6	62.3	34.2	37.0

### Test Setup and Instrumentation

**Figure 3:**
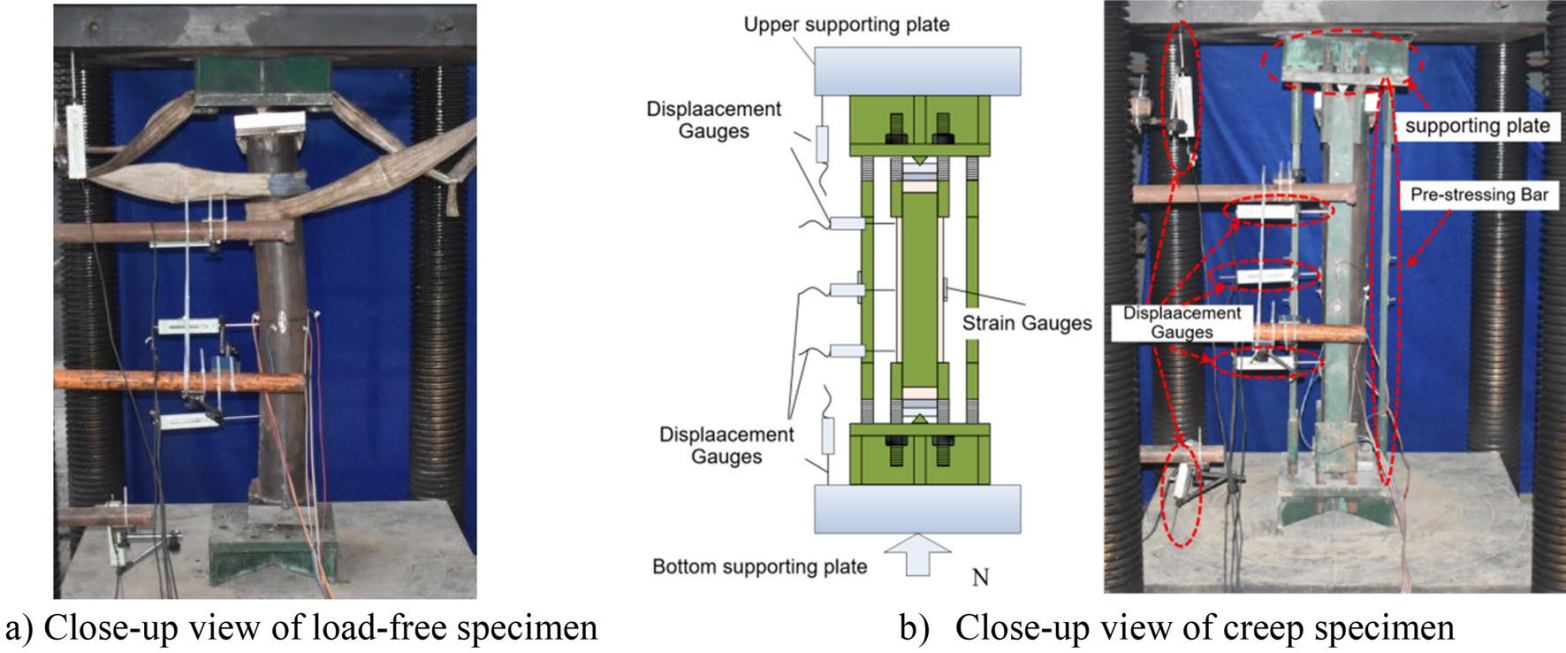
Test setup.

As shown in Fig. 3, a self-resisting loading frame was designed to perform the long-term tests on CFST specimens subjected to axial and eccentricity loads. The self-resisting loading frame was designed by referring to the designs by Geng et al.^7–11,27^. It is modeling calculation that ensures safety before fabrication. The specimens including those from Groups I, II, and III were first loaded at a concrete age of 7 days, with the long-term load (*N*_L_) for 462 days. Thereafter, the creep strain of the CFST specimens almost ceased to increase and consequently the creep tests were finished. The compression tests were conducted using an elector-hydraulic servo-controlled test machine with a maximum load capacity of 10,000 kN, as shown in Fig. 3, without removing the self-resisting loading frame. During the test, the CFST specimen was pressurized using a flat plate hinge. The test was conducted via graded loading, with each load level being 1/40 of the expected ultimate load. Further, the holding time of each load level was approximately 2 min. When the load reached approximately 0.6 times the ultimate load, each load level was reduced to 1/60 of the ultimate load. Consequently, when the damage began to appear, the test piece was loaded continuously at a slow speed until it was finally damaged.

In the compression test, four YDH-100 displacement meters (1 mm = 200 με) were used to measure both the longitudinal and transverse displacements of the CFST specimens. An automatic acquisition system (DH3816) was used to record the data of four two-way strain and displacement gauges each at the middle section of the steel tube during the test, as shown in Fig. 3.

## Test results and discussion

**Figure 4:**
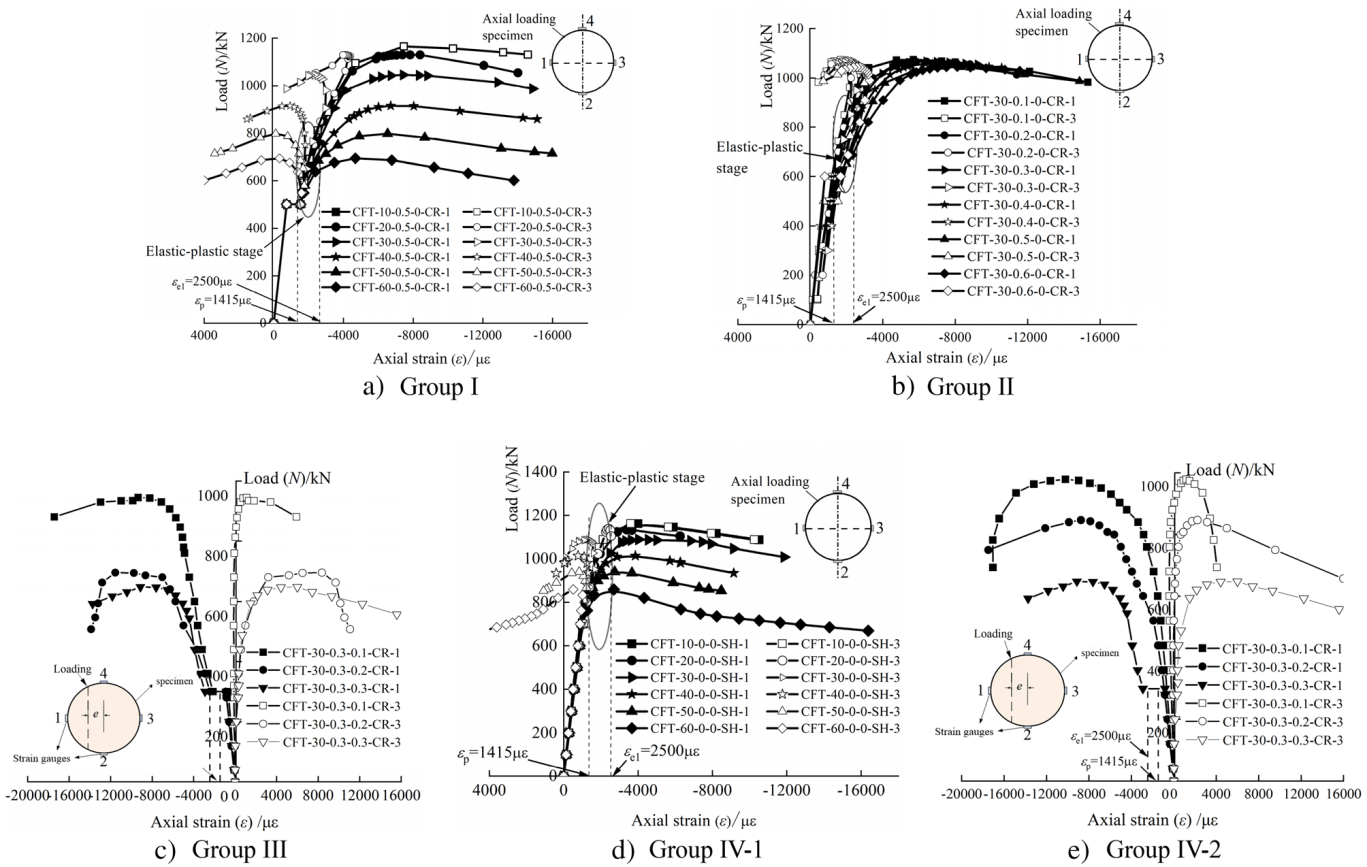
Load-axial strain curves.

The compression test results are summarized in Table 4, and the load (*N*)-axial strain (*ε*) curves of the 23 specimens are shown in Fig. 4. Based on the compression test, the following observations were obtained:

1. Compared to the specimens in Group IV, those in Groups I, II, and III yielded certain different results, as shown in Fig. 4. For the creep specimens, an initial behavior of linear elasticity was observed up to the long-term load (*N*_L_). Thereafter, a platform section was observed when being subjected to a constant long-term load; however, the creep strain increased. The new behavior of most of the creep specimens was elastic–plastic owing to the compression test being conducted without unloading the long-term load setup.
2. During the creep test under long-term load, the creep of core concrete caused the stress redistribution of the CFST composite section, the strain of steel tube increased, and the steel tube entered the elastic–plastic stage in advance. All of the specimens in Group I exhibited a proportional ultimate load (*N*_p_) during the long-term load test, as shown in Table 4 and Fig. 4. Upon the appearance of the obvious bending, the loading stopped. Moreover, the typical failure mode exhibited was buckling failure, which was caused owing to lateral bending of the creep specimens.
3. The load-strain curves comprised four distinct branches: linear ascending, platform, elastic–plastic ascending, and approximately horizontal descending branches. During the first stage, the response was similar to that of the load-free specimens. After reaching the peak, the strain was accompanied by a decrease in load. Moreover, with the increase in the slenderness and axial load ratios, the load corresponding to the plastic stage of the steel tube decreased gradually.
4. From Fig. 4d, consider the specimen of CFT-10–0-0-SH as an example. The steel tube approached the elastic–plastic stage when the loading reached approximately 870 kN and its corresponding proportional limit strain (*ε*_*p*_) was 1415 µɛ. Thereafter, the slope of curve decreased gradually. Further, the steel tube approached the plastic stage when the loading reached up to 1123 kN and its corresponding yield limit strain (*ε*_e1_) was 2500 µɛ. Subsequently, the curve increased with a smaller slope until the peak load (*N*_ue_) was 1162 kN and its corresponding maximum strain (*ε*_c_) was 3990 µɛ. Owing to the small confinement coefficient, the curve gradually decreased after the peak load. As shown in Fig. 4d, with increase in the slenderness ratio, the load corresponding to the elastic–plastic stage of the specimen gradually decreased, and the elastic-plasticity stage became shorter. Moreover, the load corresponding to the plastic stage gradually decreased. In addition, the ultimate bearing capacity of the specimen decreased with the increase in the slenderness ratio.

**Table 4:** Compression test results.

NO	Specimens	Proportional limit strain *ε*_p_ (µɛ)	Proportional limit load *N*_*p*_ (kN)	Yield limit strain *ε*_e1_ (µɛ)	Yield limit load *N*_e1_ (kN)	Maximum strain *ε*_*c*_ (µɛ)	Peak load *N*_ue_ (kN)	*k*_cr_
I	CFT-10–0.5–0-CR	1415	500	2500	812	7466	1165	1.003
	CFT-20–0.5–0-CR		500		753	7384	1129	0.991
	CFT-30–0.5–0-CR		500		764	7430	1045	0.960
	CFT-40–0.5–0-CR		500		712	6710	914	0.901
	CFT-50–0.5–0-CR		500		681	6529	798	0.849
	CFT-60–0.5–0-CR		500		648	4682	694	0.808
II	CFT-30–0.1–0-CR		654		925	5684	1074	0.986
	CFT-30–0.2–0-CR		641		879	5837	1066	0.979
	CFT-30–0.3–0-CR		566		835	6401	1067	0.980
	CFT-30–0.4–0-CR		502		810	6742	1059	0.972
	CFT-30–0.6–0-CR		600		734	8112	1044	0.958
III	CFT-30–0.3–0.1-CR		300		354	9351	946	0.966
	CFT-30–0.3–0.2-CR		300		302	11,551	695	0.822
	CFT-30–0.3–0.3-CR		300		300	9135	648	0.833
IV	CFT-10–0-0-SH		870		1123	3990	1162	/
	CFT-20–0-0-SH		868		1081	3488	1139	/
	CFT-30–0-0-SH		866		1026	4034	1089	/
	CFT-30–0-0.1-SH		572		755	7817	979	/
	CFT-30–0-0.2-SH		425		568	8746	846	/
	CFT-30–0-0.3-SH		440		566	7211	778	/
	CFT-40–0-0-SH		870		953	3858	1014	/
	CFT-50–0-0-SH		853		937	2745	940	/
	CFT-60–0-0-SH		796		856	2699	859	/

**Figure 5:**
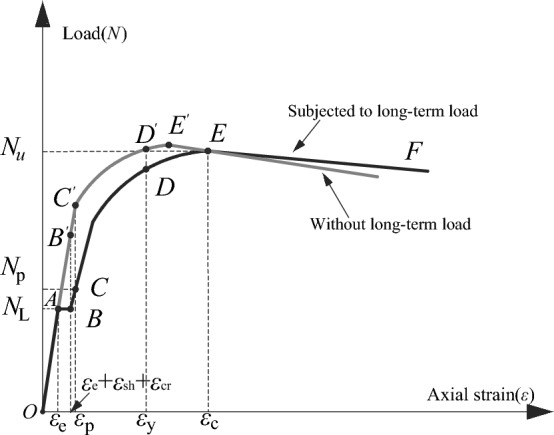
Load-axial strain curves.

**Figure 6:**
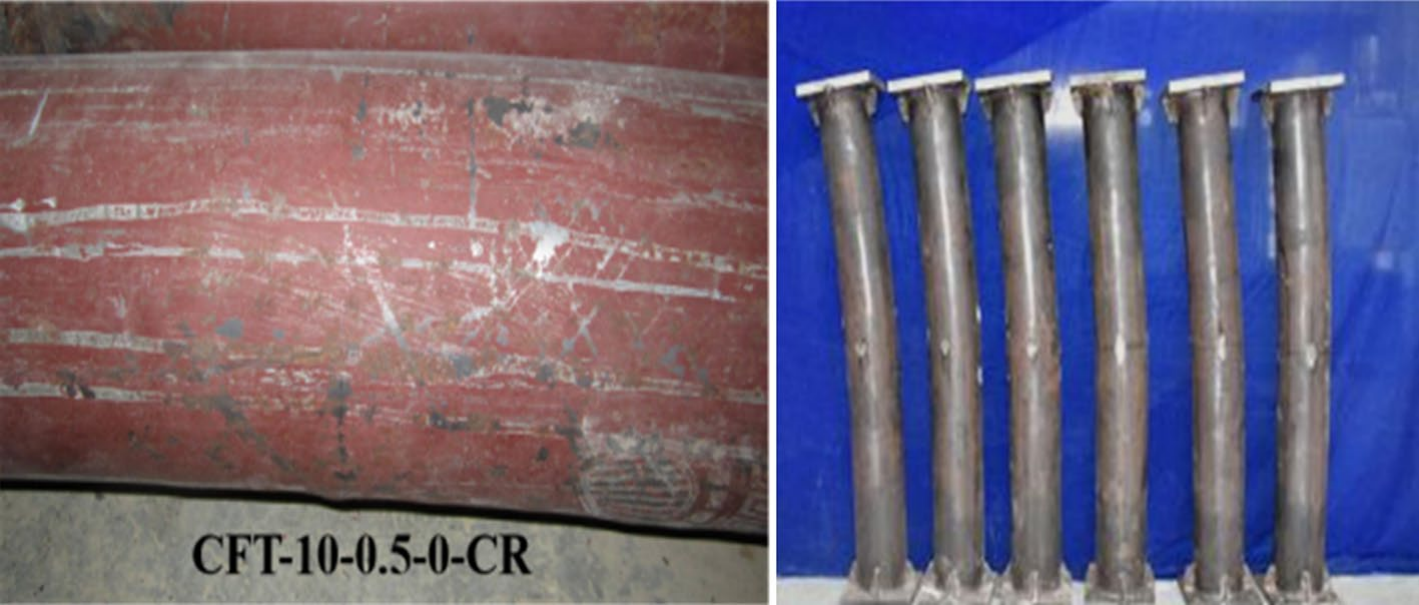
Failure mode for partial test specimens.

Based on Fig. 4, a typical load-strain curve considering the influence of creep is shown in Fig. 5. The working process is summarized as follows:

1. The *OA* section is the elastic stage (a linear ascending branch) caused by the long-term load effect at the beginning of the test. It may appear as an elastic–plastic branch when the axial load ratio is larger. The load corresponding to point *A* is the long-term load (*N*_L_), and the corresponding strain is the initial elastic strain (*ε*_*e*_).
2. Section *AB* indicates the stage of increased shrinkage and creep deformation under long-term load. Because of the effect of shrinkage and creep, the axial strain of the CFST column increased until the end of the long-term load test (point *B* on the curve). The corresponding load was still *N*_L_. However, the corresponding strain increased to *ε*_*e*_ + *ε*_*sh*_ + *ε*_*cr*_, where *ε*_*sh*_ and *ε*_*cr*_ are the shrinkage and creep strains of the CFST column, respectively.
3. Section *BC* is the elastic stage (or possibly elastic–plastic stage), wherein the specimen was tested until failure after finishing the long-term load test. Point *C* corresponds to the proportional limit load (*N*_p_) of the steel tube and the strain of steel tube is proportional to the limit strain (*ε*_*p*_). Here, the load is less than the load corresponding to the load-free member (corresponding to the point *C*' in Fig. 5).
4. Section *CD* is the elastic–plastic stage. Owing to the sustained load applied on the composite cross section for long term, the stress of the steel tube increases with time, whereas that of the core concrete reduces. The axial force distribution ratio between the steel tube and core concrete changes constantly. Upon the completion of the creep test, the stress of steel tube was larger than that of load-free specimen. Consequently, the applied loading was reloaded on the creep specimens after the creep test and the core concrete was re-stressed. Thus, the stress of steel tube increases constantly such that it enters the elastic–plastic stage in advance (i.e., the load corresponding to point *C* in the Fig. 5 is less than that at point *C*'). Thereafter, the load-strain relationship gradually deviates from the straight line to form a transition curve (elastic–plastic stage). Compared with the load-free specimens, the range of the elastic–plastic stage for creep specimens is larger (i.e., the range of *CD* section is larger than that of *C*'*D*' section, as shown in Fig. 5). The tangent modulus decreased continuously after the stress of steel tube entered the elastic–plastic stage. Whereafter, the force of core concrete increased with the increasing loading. Then, its Poisson's ratio exceeded that of the steel tube (i.e., an increasing interaction force referred to as the confining force between the steel tube and core concrete appeared; both of them were in a triaxial stress state (such as specimen CFT-10–0.5–0-CR)). Before the load up to point *D* (i.e., the steel tube was yielded, the corresponding strain was *ε*_*y*_), a neat oblique shear slip line appeared on the outer surface of the steel tube, as shown in Fig. 6.
5. Section *DE* is the plastic strengthening stage. After point *D*, the steel tube entered the fully plastic stage, wherein the increasing load was undertaken by core concrete, its lateral deformation increased rapidly, and the hoop stress of the steel tube increased (i.e., the confining force increases). Therefore, the core concrete was subjected to lateral pressure and improved its bearing capacity. The improvement in the bearing capacity of core concrete compensates for and exceeds the decrease in the longitudinal internal force of steel tube, thereby forming a plastic strengthening section until the member is destroyed. Compared with the load-free members, owing to creep, the steel tube entered the plastic stage relatively early, the stress of core concrete was relatively lagging behind, the confinement effect of steel tube was delayed, and the ultimate load of load-free member was also relatively lagging behind. Further, the maximum load corresponding to point *E* is defined as the ultimate (or stability) bearing capacity, expressed as *N*_u_, and the corresponding strain is the ultimate strain (*ε*_c_).
6. Section *EF* is the failure descent stage. For a short column (i.e., specimen CFT-10–0.5–0-CR), when the loading up to point *E*, the specimen appears as a local buckling and finally shear failure occurs. At this time, with the increase in strain, the load does not increase further; however, a descending curve appears. In case of medium-length columns, the specimen is unstable and exhibits longitudinal bending when destroyed, as shown in Fig. 6.

The observations of the test results indicate that the creep behavior decreases the stiffness of the CFST columns. During the 462 days of long-term loading, the applied loading was invariable yet the strain of the composite columns increased continuously. According to the Hooke's law, that is *σ* = *Eε*, when the average stress (*σ*) in the composite cross section is invariable, the strain (*ε*) of steel tube increases with time. Consequently, the Young modulus (*E*) of the composite cross section must be decreased. Thus, the stability bearing capacity decreases with the reduction in stiffness.

### Influence of slenderness ratio

**Figure 7:**
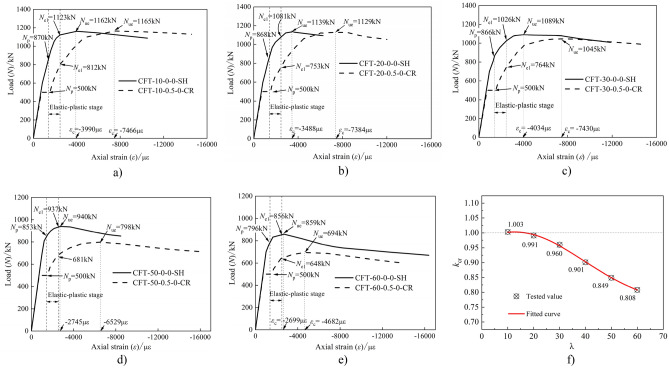
Comparisons of *N*-*ε* curve between Group I and IV.

Figure 7 presents a comparison of the load (*N*)- strain (*ε*) curves of specimens subjected to long-term load (i.e., specimens for Group I) and the specimens that were load-free during the long-term load tests (i.e., specimens for Group IV). The ratio of the ultimate loads of those specimens under long-term load to that of the load-free specimens has been depicted by the parameter *k*_cr_ (i.e., the creep reduction coefficient) listed in Table 4. From these results it can be observed that:

1. Compared with the load-free specimens, the stiffness of the specimens after long-term load test is smaller, and the axial strain corresponding to the stability bearing capacity is larger. For example, the maximum strain (*ε*_*c*_) of the steel tube corresponding to the peak load (*N*_u_) of the specimen CFT-40–0-0-SH was 3858 µɛ, and that of CFT-40–0.5–0-CR (i.e., after long-term load test) was 6710 µɛ.
2. The stability bearing capacity of the specimens under long-term load is lower than that of the load-free specimens. From Table 4, it can be observed that for the specimen with a slenderness ratio of 10 (i.e., specimen CFT-10–0.5–0-CR and CFT-10–0-0-SH), the peak load of the specimen subjected to long-term load was higher than the specimen that was unloaded. This result was also observed by Yuyin Wang^27^. The larger the slenderness ratio, the more obvious the effect of long-term load on the stability bearing capacity of the CFST specimens. With the increase in the slenderness ratio, the peak load was lower and the creep reduction coefficient (*k*_cr_) reduced. Moreover, the peak load of specimen CFT-60–0.5–0-CR was approximately 20% lower than its contrast specimen (i.e., specimen CFT-60–0-0-SH).
3. For the specimens subjected to long-term load, stress redistribution occurred in the section of the CFST composite section. Maintaining the long-term load as a constant value, the stress of the steel tube increased and that of the core concrete decreased during the creep test. Thus, in the compressive test, the steel tube approached the yield stress in advance compared to the contrast specimen, whereas the core concrete delayed the stress. Such phenomena could relatively prolong the process of the steel tube from yield to failure (strength failure or instability failure).

### Influence of axial load ratio

**Figure 8:**
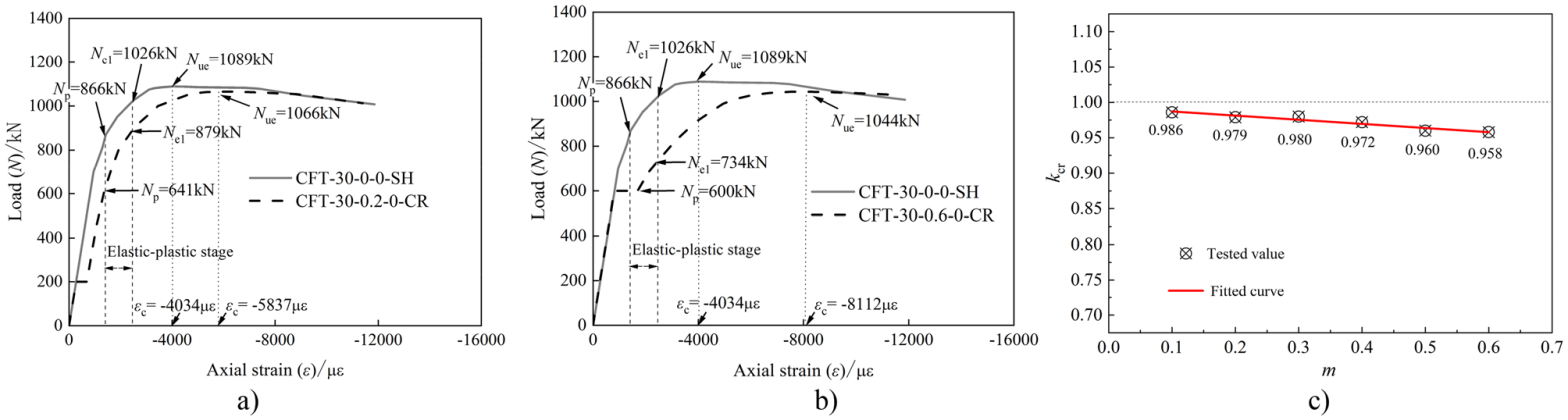
Comparisons of *N*-*ε* curve between Group II and III.

The load (*N*)-strain (*ε*) curves of the specimens subjected to sustained loading (i.e., specimens for Group II) and the specimens that were load-free during the long-term tests (i.e., specimen CFT-30–0-0-SH) are shown in Fig. 8.

All the specimens collapsed in a instability failure mode, indicating that time did not influence the mode of failure. The figure shows that:

1. Compared with the specimen CFT-30–0-0-SH, the axial strain corresponding to the peak load of the CFST specimen subjected to long-term load was larger. Further, the difference increased with the increase in the axial load ratio (*m*).
2. The stability bearing capacity of the specimens subjected to long-term load was lower than that of the companion load-free specimen (i.e., specimen CFT-30–0-0-SH). The peak load of the creep specimens with the axial load ratio (*m*) from 0.1–0.6 was gradually decreased. Further, the ratio of the peak load of the creep specimen to that of the companion load-free specimen was in the range of 0.986–0.958, which appeared as a linear variation, as shown in Fig. 8 c).
3. The larger the axial load ratio, the smaller the load corresponding to the elastic–plastic and plastic stages of the CFST creep specimen. Further, the larger the axial load ratio, the larger the strain of the steel tube under the same load, and the more obvious the reduction in the slope of the elastic–plastic stage. For the ultimate strain corresponding to the peak load of the creep specimen, the larger the axial load ratio, the greater the value.
4. The strain of the tensile zone corresponding to the cross section of the specimen under sustained load was larger than that of the companion specimen. This indicates that the creep effect caused by long-term load increased and decreased the stress of the steel tube and tensile zone, respectively.

### Influence of eccentricity ratio

**Figure 9:**
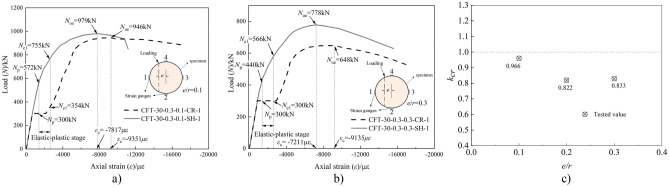
Comparison of load-axial strain curves between eccentricity and comparison specimens.

Figure 9 shows the load (*N*)-strain (*ε*) curves of the specimens in Group III (i.e., creep specimens with the specimen parameter of eccentricity ratio (*e*/*r*)) and the comparison load-free specimens. Compared with the load-free specimen, the load (*N*)-strain (*ε*) curve of the creep specimen exhibited a displacement platform segment owing to the long-term load. The final failure mode of all the specimens is instability failure. When the creep specimen reaches the stability bearing capacity, the maximum strain (*ε*_c_) is obviously greater than that of the load-free specimen. Compared with CFT-30–0-0.1-SH, CFT-30–0-0.2-SH, and CFT-30–0-0.3-SH, the stable bearing capacity of CFT-30–0.3–0.1-CR, CFT-30–0.3–0.2-CR, and CFT-30–0.3–0.3-CR decreased by 3.4, 17.8, and 16.7%, respectively.

As shown in Fig. 9a, when specimen CFT-30–0-0.1-SH reached its ultimate load of 979 kN, the ultimate strain was 7817 με. Further, when the specimen CFT-30–0.3–0.1-CR reached its ultimate load of 946 kN, the ultimate strain reached 9351 με. Compared with specimen CFT-30–0-0.1-SH, the maximum strain (*ε*_c_) of specimen CFT-30–0.3–0.1-CR near the loading side increased by 19.6%. As shown in Fig. 9, when the long-term load was unchanged, the eccentricity was larger, the creep effect on core concrete near the load side was more pronounced, and the redistribution of internal forces on the section was obvious. Moreover, the axial strain of the steel tube near the load side increased faster.

## Finite-element analysis

In practical engineering, the influence of long-term load on CFST structure is continuous; that is, the increase and decrease in steel tube and core concrete stresses caused by creep, respectively, eventually affect its stability bearing capacity. Therefore, to simulate the real stress state more accurately, the influence of long-term load on the stability bearing capacity of the CFST column is considered without unloading the long-term load. Further, the one-time loading failure is directly based on the long-term load.

### Calculation method

The finite element software ANSYS was used to establish the mechanical analysis model of a CFST column considering the influence of long-term load. The entire process of the load-axial strain relationship curve of the CFST column considering the influence of long-term load was calculated. The calculation was divided into the following three stages:

The first stage was the application of the long-term load, and the stress–strain relationship curves of steel tube and core concrete did not incorporate the effect of the long-term load.

The second stage was the development stage of creep deformation. Herein, the long-term load remained unchanged, the strain of concrete filled steel tube increased continuously, the stress of steel tube increased gradually, and the stress of core concrete decreased gradually until the end of creep deformation. Here, the stress increment of the steel tube and the stress reduction of the core concrete were calculated based on the subroutine developed by Chen and Shrestha^33^.

**Figure 10:**
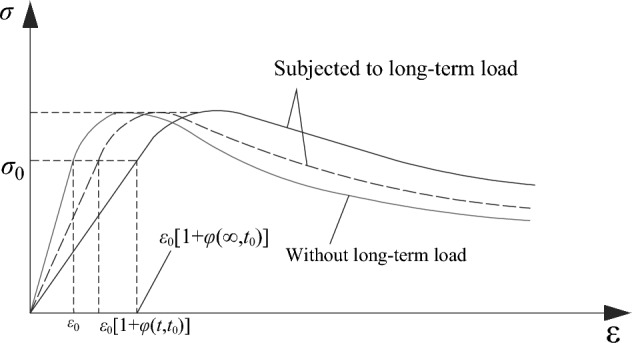
Constitutive relation curve of concrete considering the influence of long-term load.

The third stage was the stage of continuous loading without unloading until the failure of the CFST column following the completion of the creep test. The core concrete in this stage adopted the stress–strain relationship curve considering the influence of the long-term load, as shown in Fig. 10. This study employed the constitutive relationship of the core concrete with the confinement proposed by Han^30^ to analyze the stability bearing capacity of CFST columns.

As shown in Fig. 10, the strain multiplied by [1 + *φ*(*t*,*t*_0_)] was used to modify the stress–strain model of core concrete. It is assumed that the long-term load does not affect the strength of the core concrete; rather, it only affects the change in its strain. This yielded the relationship between the strain (*ε*_*l*_) subjected to long-term load and the strain (*ε*) without the long-term load, as expressed in Eq. (1).

1 $$\varepsilon_{l} = \left[ {1 + \phi (t,t_{0} )} \right] \cdot \varepsilon + \varepsilon_{sh}$$

where *ε*_*l*_ is the strain of core concrete subjected to long-term load, *ε* is the strain of core concrete without subjecting to long-term load, *φ*(*t*,*t*_0_) is the creep coefficient of core concrete, and *ε*_*sh*_ is the shrinkage strain of concrete filled in the steel tube.

The beam element (i.e., double element method, the steel tube and the core concrete are established, respectively) was used to model the CFST members. The spatial elastic–plastic beam element BEAM188 can simulate both short and thick and slender beams and columns. Further, they can also simulate the cross section of any shape. In this study, the finite element model of concrete filled steel tube structure was simulated using the BEAM188 element.

### Comparison between experimental and predicted results

For the tested CFST specimens, the following sections present a comparison of the prediction of load-axial strain response with the test results.

**Figure 11:**
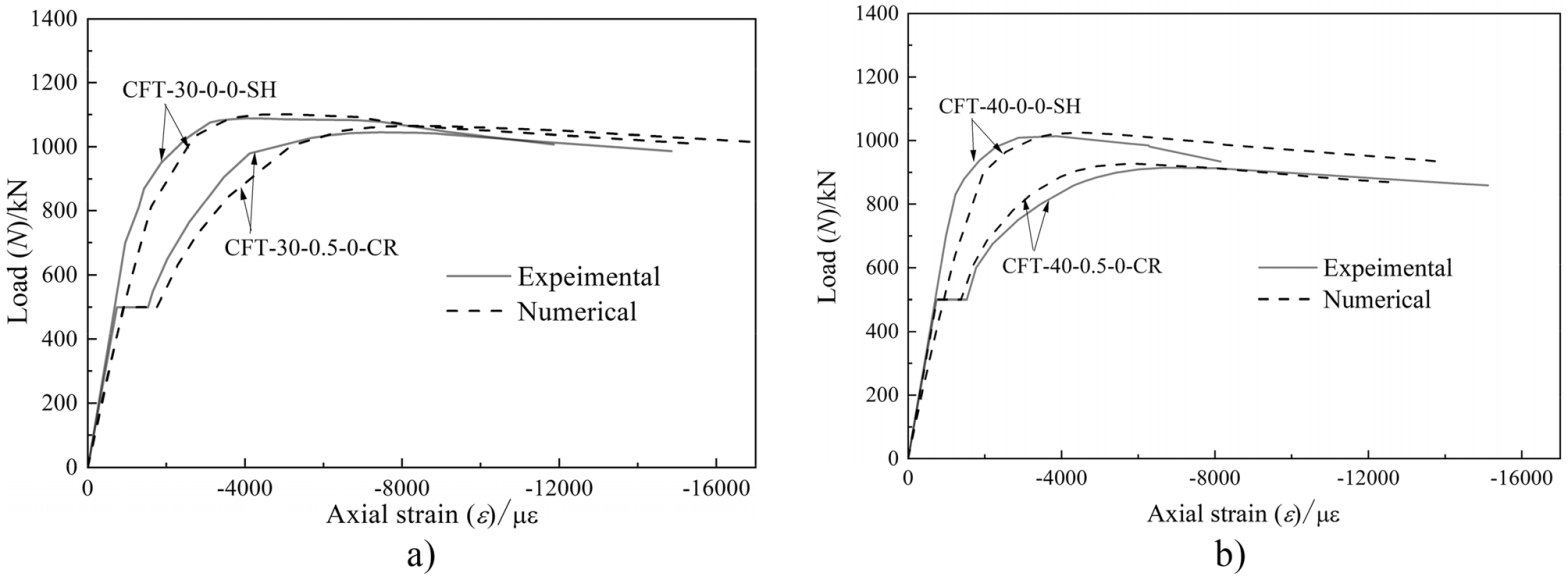
Comparison between test and numerical results of specimens.

The load-axial strain curves for the partial CFST columns are shown in Fig. 11. The ultimate bearing capacity (*N*_uc_) predicted by finite element analysis is presented in Table 1. The predicted load-axial strain curves of the creep specimens were obtained from the proposed model. Whereas, for the load-free companion specimens, the short term plasticity constitutive model by Han^30^ was applied to simulate their load-axial strain behavior as they were not subjected to long-term load.

For the creep coefficient of the core concrete [*φ*(*t*,*t*_0_)], the ACI 209R-92 creep model was used to perform calculations with the experimental parameters^34^. Because of the core concrete infilled steel tube, moisture exchange was prevented; the relative humidity of concrete was assumed as 90% for calculating *φ*(*t*,*t*_0_).

Figure 11 and Table 1 show that the mean and standard deviations of *N*_ue_/*N*_uc_ were 0.991 and 0.011, respectively. As evident, the finite element model can well simulate the mechanical behavior of the entire process for the CFST column considering the effect of long-term load.

**Figure 12:**
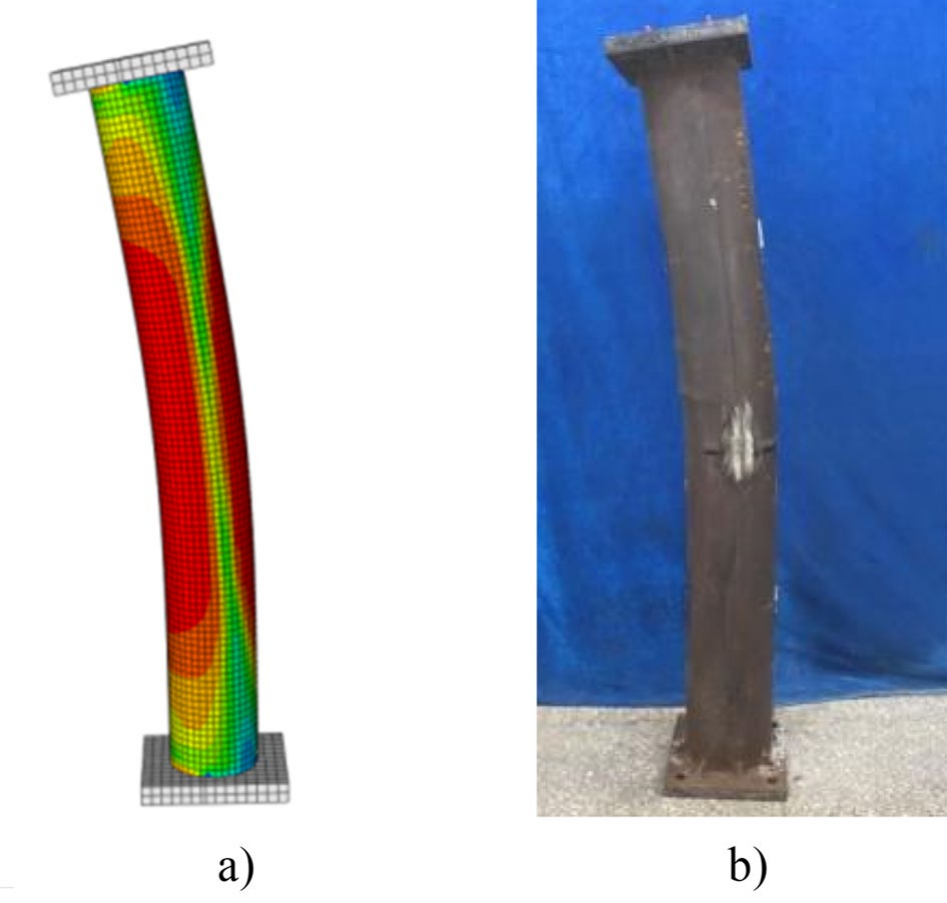
Failure pattern comparison between finite element method and test results.

The failure pattern comparison between finite element results and test is presented in Fig. 12. The cloud chart of the FE results indicate that the finite element method proposed in this study is consistent with the experimental phenomena. It is suitable for the parametric analysis to evaluate the creep effect on the stability bearing capacity of CFST columns. As shown in Figs. 11 and 12, the deformation or failure patterns between the test specimen and finite element results were similar. The lateral deformation at the mid-height section of the CFST specimens increased rapidly before the applied load reached to peak load. Further, the local buckling occurred at the mid-height section of the steel tube, as shown in Fig. 12. Here, the specimen experienced buckling failure and exhibited longitudinal bending when destroyed.

According to the experimental results, the stability bearing capacity of the CFST creep specimens decreases with the increase in the slenderness and axial load ratios. In this study, to simplify parameters during parameter analysis, the influence of axial load ratio (*m*) was converted into the creep stress degree (*β*_cr_) considering the steel ratio and concrete component materials^35^. Further details ca be found in 35. According to literature 5, the parameters required to analyze the stability bearing capacity of CFST creep and load-free specimens are as follows: creep stress degree (*β*_cr_) range of 0.1–0.7, slenderness ratio (*λ*) range of 10–160, eccentricity ratio (*e/r*) range of 0–1, concrete strength grade range of C30–C60, and three commonly used low carbon steel of Q235, Q345, and Q390. A total of 252 components were calculated to analyze the influence coefficient *k*_cr_ ($k_{{{\text{cr}}}} = N_{{{\text{max}}}}^{{\text{c}}} /N$, where $N_{\max }^{c}$ is the ultimate bearing capacity of CFST members subjected to long-term load and *N* is the ultimate bearing capacity of CFST load-free members).

**Figure 13:**
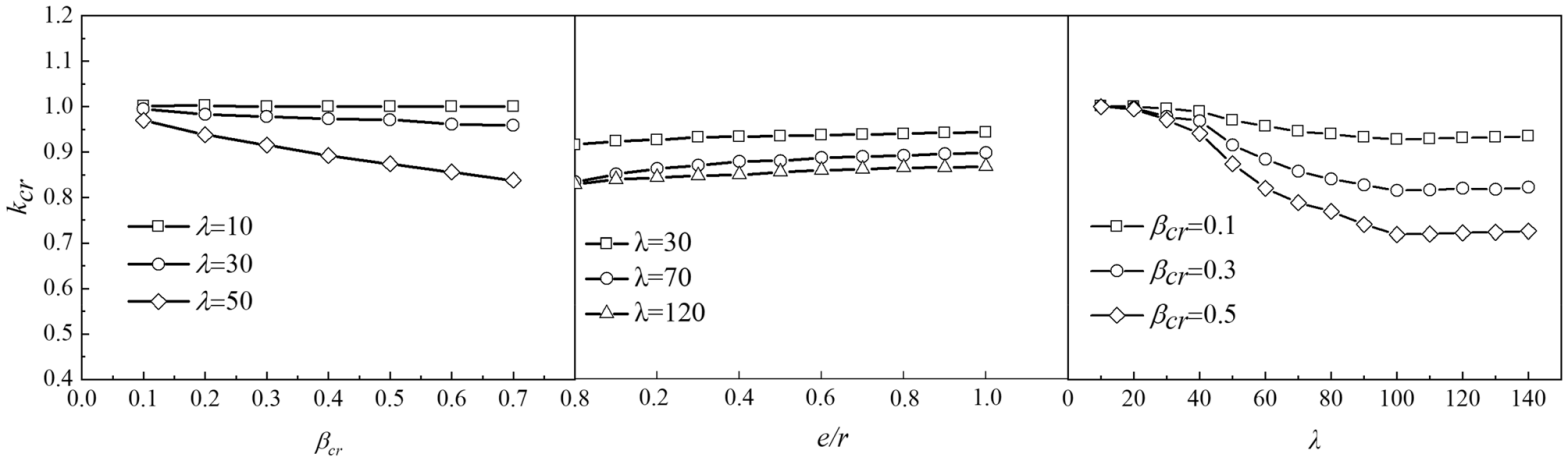
Relationship between *λ*, *β*_cr_, *e*/*r*, and *k*_cr_.

Figure 13 shows the relationship between *λ*, *β*_cr,_ and *k*_cr_. Creep effects decrease the stability bearing capacity of CFST columns. As evident, *k*_cr_ decreases with the increase in *λ* and *β*_cr_ when λ is less than 100; *k*_cr_ gradually flattens out with the increase in λ, and has a tendency to increase. The primary reason for this phenomenon is that in case of a small slenderness ratio of the CFST member, the mid-span section is fundamentally in the full section compression state. The stress increment of the steel tube caused by the long-term load results in the member entering the plastic stage in advance, which affects the stability bearing capacity of the member. However, when the slenderness ratio is relatively large, the stability problem of the component itself is more prominent. Moreover, the influence of the long-term load on its stable bearing capacity decreases instead. Thus, the effect of *β*_cr_ on *k*_cr_ indicated that *k*_cr_ decreased linearly with the decrease in *β*_cr_.

According to the statistics presented in the literature 34, the maximum *β*_cr_ of concrete filled steel tube arch bridge is 0.29 and its corresponding value of *k*_cr_ is 0.812, as shown in Fig. 13. Thus, the stability bearing capacity of the CFST members decreased by approximately 20% after long-term load, which cannot be ignored. Therefore, it is suggested that bridge designers should consider the reduction of the bearing capacity caused by long-term load when performing calculations.

Figure 13 shows that, with the increase in load-holding eccentricity ratio (*e*/*r*), the creep influence coefficient (*k*_cr_) exhibited an increasing trend; that is, the influence of the long-term load on stability bearing capacity reduced. When *e*/*r* reached 0.6, *k*_cr_ gradually became stable. When the slenderness ratio (*λ*) was 30, *k*_cr_ was approximately stable at 0.94. For slenderness ratio (*λ*) of 70, *k*_cr_ was approximately stable at 0.89. Whereas, for slenderness ratio (*λ*) of 120, *k*_cr_ was approximately stable at 0.86.

### Practical algorithms and suggestions

As shown in Fig. 13, the creep influence coefficient (*k*_cr_) was affected by the change in the slenderness ratio (*λ*), creep stress degree (*β*_cr_), and eccentricity ratio (*e*/*r*). Thus, based on the parameter analysis and tested data, to construct a two-stage function with a slenderness ratio (*λ*) of 40 as the boundary, the relationship between *k*_cr_ and creep stress degree (*β*_cr_) was obtained as linear, which is expressed as the first term in Eq. (2). However, with the increase in the slenderness ratio, its influence was also intensified. The second term in Eq. (2) indicates the influence of the slenderness ratio (*λ*) and creep stress degree (*β*_cr_). Further, the third term in Eq. (2) indicates the effect of the slenderness ratio (*λ*) and eccentricity ratio (*e*/*r*). Via regression fitting of the finite element calculation results of the 252 components, the creep influence coefficient (*k*_cr_) considering the influence of long-term load is expressed as Eq. (2) :

2 $$k_{cr} = \left\{ \begin{gathered} (1 - 0.35\beta_{cr} ) \cdot \frac{{1 + 0.45\beta_{cr} }}{{1 + \beta_{cr} \lambda_{0}^{2} }} \cdot [1 + 0.28\lambda_{0} (1 - c)]\,\,\,\,\,\,\,\,\,\,\,\,\,\,\,\,\left( {\lambda_{0} \le 0.4} \right) \hfill \\ (1 - 0.35\beta_{cr} ) \cdot \left( {a_{1} \lambda_{0}^{2} + a_{2} \lambda_{0} + a_{3} } \right) \cdot [1 + \frac{1 - c}{{8.1 + 5.2\lambda_{0}^{2} }}]\,\,\,\,\,\,\,\left( {\lambda_{0} \ge .4} \right) \hfill \\ \end{gathered} \right.$$

where a_1_ = 0.84 *β*_cr_ + 0.05, a_2_ = -1.93*β*_cr_-0.1, a_3_ = 0.87*β*_cr_ + 1.04, *λ*_0_ = *λ*/100, *λ* = 10 ~ 160, *c* = (1 + *e*/*r*)^-2^, and *β*_cr_ is the creep stress degree with a range of 0.1–0.7.

An experimental database was compiled to evaluate Eq. (2). The database included 49 test data from another study and the test in this study, as summarized in Table 5. Table 5 presents the steel ratio of the test specimens, eccentricity ratio (*e*/*r*), slenderness ratio (*λ*), measured peak load (*N*_ue_ ), and predicted peak load (*N*_uct_) obtained using Eq. (2) (where *k*_cr_ is predicted using Eq. (2)). The mantissa of component number "e" and "a" in reference 24 in Table 5 represent the eccentric compression and axial compression members, respectively. The *N*_uct_ value was obtained using the formula *N*_uct_ = *ψ*_e_·*ψ*·*k*_cr_·*N*_0_; where *ψ*_e_ is the eccentric influence coefficient, *ψ* is the stability coefficient of slenderness ratio, *k*_cr_ is the creep influence coefficient, and *N*_0_ is the design axial compressive strength predicted from the national standard GB 50,923–2013^31^. Table 5 also summarizes the ratio of the measured peak load (*N*_ue_ ) to the predicted peak load (*N*_uct_). As evident, the mean ratio of the measured to predicted peak load was 1.036. The corresponding variance was 0.175. Furthermore, the *N*_uct_ calculated using Eq. (2) exhibited good mean prediction quality and high coincidence.

**Table 5:** Comparisons of predicted and measured values of influence factor.

Reference	Specimen	Steel ratio (*α*)	Eccentricity ratio (*e*/*r*)	Slenderness ratio (λ)	Measured peak load (*N*_ue_ )/kN	Predicted peak load from Eq. (2) (*N*_uct_)/kN	*N*_ue_/ *N*_uct_
Chen^36^	CR-C40-0.2–14	0.057	0.2	30	750	733	1.023
	CR-C50-0.2–14	0.057	0.2	30	944	1041	0.907
	CR-C40-0.2–28	0.057	0.2	30	844	734	1.150
	CR-C50-0.2–28	0.057	0.2	30	989	1041	0.950
Tan and Qi^24^	1e	0.0716	0.40	17	449	367	1.223
	2e	0.0740	0.55	17	372	320	1.163
	4e	0.0855	0.80	17	284	281	1.011
	5e	0.0687	0.40	17	456	364	1.253
	8e	0.0719	0.80	17	269	262	1.027
	9e	0.1595	0.40	17	726	544	1.335
	10e	0.1620	0.55	17	573	475	1.206
	12e	0.1645	0.80	17	460	392	1.173
	14e	0.1592	0.55	17	650	469	1.386
	1a	0.066	0	16	560	582	0.962
	2a	0.072	0	16	636	594	1.071
	3a	0.072	0	16	569	599	0.950
	4a	0.077	0	16	652	573	1.138
	5a	0.077	0	16	641	573	1.119
	6a	0.077	0	16	622	573	1.086
	7a	0.082	0	16	660	586	1.126
	8a	0.092	0	16	780	656	1.189
	9a	0.092	0	16	580	656	0.884
	10a	0.093	0	16	700	618	1.133
	11a	0.093	0	16	662	619	1.069
	12a	0.099	0	16	720	636	1.132
	13a	0.151	0	16	975	828	1.178
	14a	0.151	0	16	1076	788	1.365
	15a	0.157	0	16	954	804	1.187
	16a	0.157	0	16	997	805	1.239
	17a	0.157	0	16	967	845	1.144
	18a	0.167	0	16	1047	873	1.199
	19a	0.174	0	16	950	894	1.063
	20a	0.172	0	16	1020	888	1.149
	21a	0.173	0	16	869	851	1.021
	22a	0.178	0	16	1067	905	1.179
This paper	CFT-10–0.5–0-CR	0.057	0	10	1165	1408	0.827
	CFT-20–0.5–0-CR	0.057	0	20	1129	1351	0.836
	CFT-30–0.5–0-CR	0.057	0	30	1045	1260	0.829
	CFT-40–0.5–0-CR	0.057	0	40	914	1145	0.798
	CFT-50–0.5–0-CR	0.057	0	50	798	992	0.804
	CFT-60–0.5–0-CR	0.057	0	60	694	850	0.816
	CFT-30–0.1–0-CR	0.057	0	30	1074	1281	0.838
	CFT-30–0.2–0-CR	0.057	0	30	1066	1279	0.833
	CFT-30–0.3–0-CR	0.057	0	30	1067	1274	0.838
	CFT-30–0.4–0-CR	0.057	0	30	1059	1269	0.835
	CFT-30–0.6–0-CR	0.057	0	30	1045	1252	0.835
	CFT-30–0.3–0.1-CR	0.057	0.1	30	946	1120	0.845
	CFT-30–0.3–0.2-CR	0.057	0.2	30	695	976	0.712
	CFT-30–0.3–0.3-CR	0.057	0.3	30	648	864	0.750

Through the above comparative analysis, it is evident that the formula for calculating the creep influence coefficient of the CFST column proposed in this study can better reflect the influence of the long-term load on the stable bearing capacity of the CFST column. Furthermore, the formula can serve as a reference for the calculation of CFST structure design.

## Summary and conclusions

After 462 days of long-term load test, a stable bearing capacity test was conducted for CFST specimens subjected to long-term load as well as the comparison specimens that were load-free. The load (*N*)-axial strain (*ε*) curve of the entire process was analyzed, and the influence of three parameters: slenderness ratio (*λ*), axial load ratio (*m*), and eccentricity ratio (*e*/*r*) on the stable bearing capacity of the CFST specimen after long-term load subjugation was analyzed. The creep reduction coefficient (*k*_cr_) algorithm of the stable bearing capacity of the CFST column considering the effect of long-term load was compared and analyzed, and the data of 49 test specimens were collected to perform evaluations. Consequently, a practical algorithm was proposed. Based on comparisons with the measured data, the following conclusions were drawn:

1. The results showed that the stability bearing capacity of the CFST columns can decrease by up to approximately 20% after 462 days of sustained loading. The failure pattern of all the creep specimens was similar to that of the load-free specimens. The larger the slenderness ratio, the more obvious the effect of the long-term load on the stability bearing capacity of the CFST specimens. With the increase in the slenderness ratio, the peak load became lower and the creep reduction coefficient reduced. For specimens with a parameter of axial load ratio, the ratio of the peak load of the creep specimen to that of the companion load-free specimen was in the range of 0.986–0.958, and appeared as a linear variation. With increase in the eccentricity, the axial strain of the steel tube near the load side increased faster, and the stability bearing capacity reduced.
2. Compared with the load-free CFST column, the corresponding load decreased when the steel tube entered the elastic–plastic and plastic phases after the action of long-term load. In contrast, the corresponding strain increased when the member reached the maximum load. Further, with the increase in the slenderness ratio (*λ*) and axial load ratio (*m*), the influence of the long-term load on the stable bearing capacity of the CFST column was greater, with the influence of the former being greater than that of the latter.
3. Based on the experimental data, the formula for calculating the reduction coefficient (*k*_cr_) of the stable bearing capacity of a CFST column considering the effect of long-term load was proposed in this study. The influencing parameters were simplified into slenderness ratio *λ*, creep stress *β*_cr_, and eccentricity ratio *e/r*, which greatly simplified the calculation workload. The established finite element model well simulated the mechanical behavior of the CFST columns under long-term loads, and the finite element analysis results were verified by the experimental results. The parameter analysis results showed that when *λ* was less than 100, *k*_cr_ decreased with the increase in both *λ* and *β*_cr_. Further, *k*_cr_ gradually tended to be flat with the increase in *λ*, and exhibited an increasing trend when *λ* was larger than 100. In contrast, *k*_cr_ decreased linearly with the increase in *β*_cr_. Moreover, with the increase in *e*/*r*, *k*_cr_ again exhibited an increasing trend; that is, the influence of the long-term load on the stability bearing capacity became smaller.
4. The results and suggestions in this study were based on the laboratory condition and the number of specimen and database were limited. Moreover, additional experimental parameters will be considered in future research such as the concrete grade, concrete age, and steel ratio because all these factors may affect the stability bearing capacity of CFST columns subjected to a long-term load.

## Data Availability

The datasets generated and/or analysed during the current study are not publicly available due to the privacy of individuals that participated in the study but are available from the corresponding author on reasonable request.
